# Supplementary material for: Factors Influencing Mammographic Density in Asian Women: A Retrospective Cohort Study in the Northeast Region of Peninsular Malaysia
Source: Diagnostics (Basel). 2022 Mar 30;12(4):860. doi: 10.3390/diagnostics12040860 (PMC9032698; doi:10.3390/diagnostics12040860)
Supplement: Supplementary file 1 [file diagnostics-12-00860-s001.zip › diagnostics-1647193-supplementary.pdf]

Convergence of the algorithm in multiple imputations

The algorithm was considered converged as the line in the plots were intermingled and free of trend.

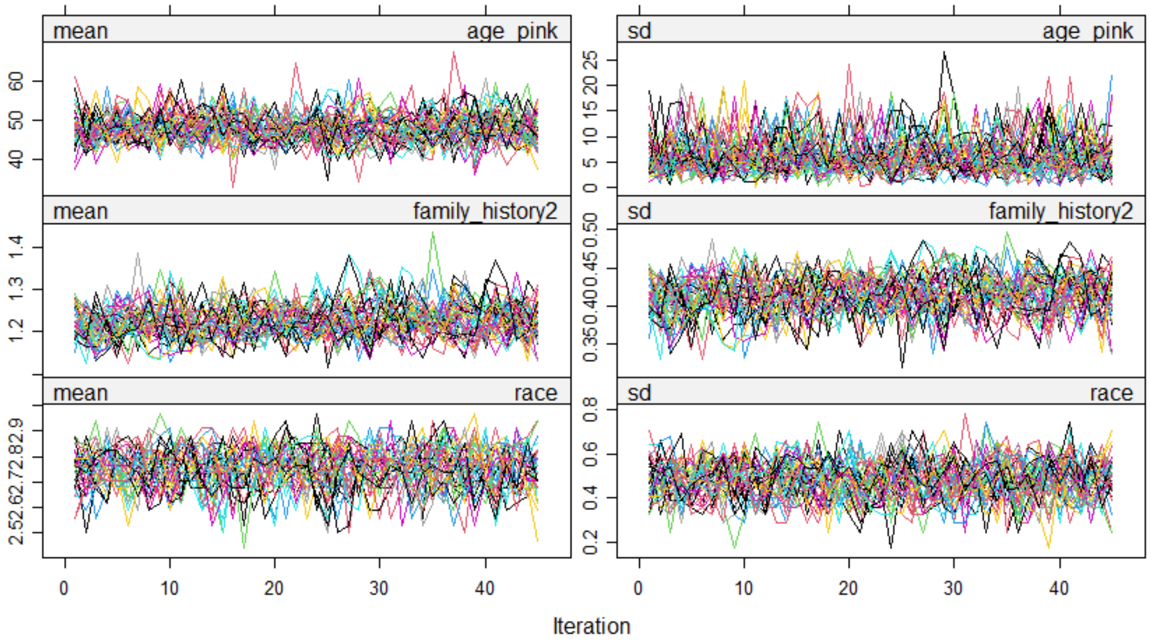

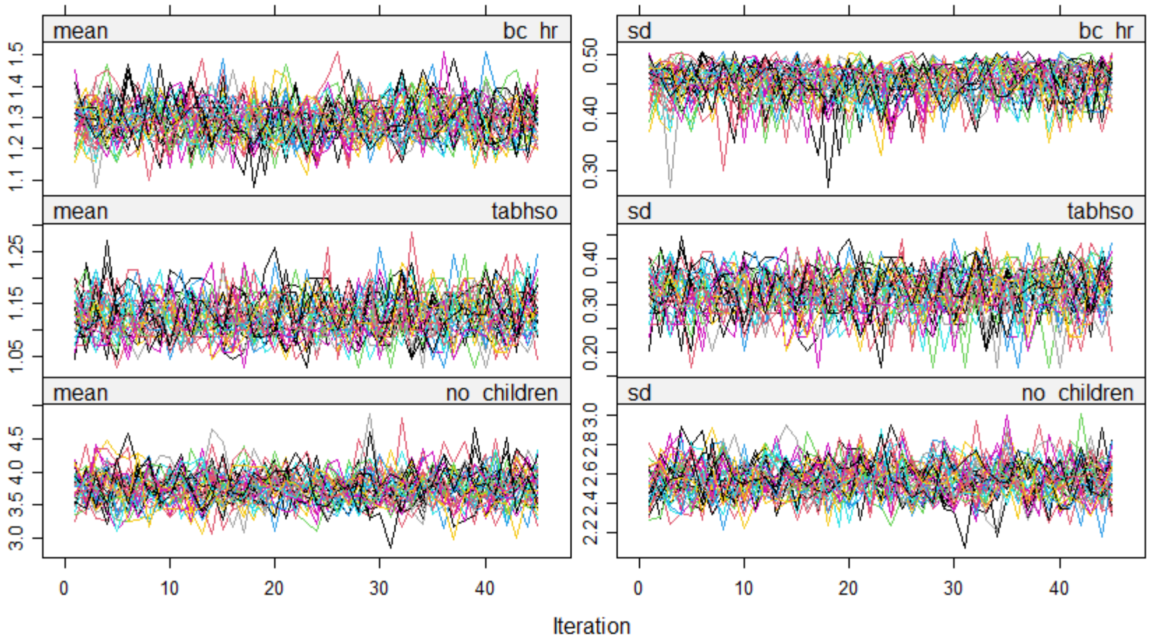

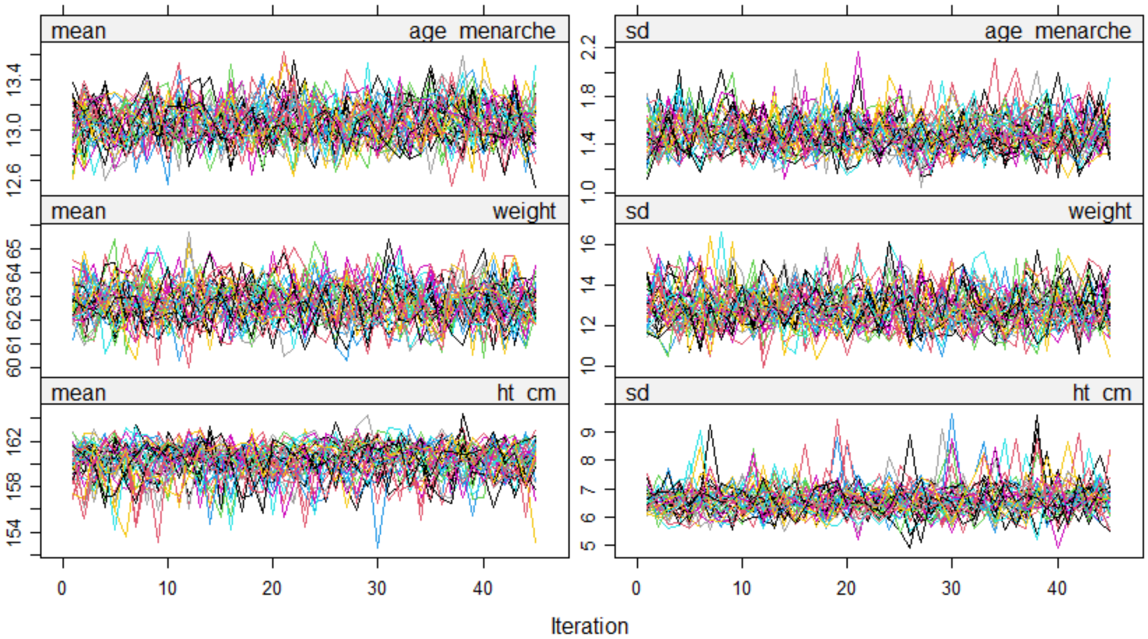

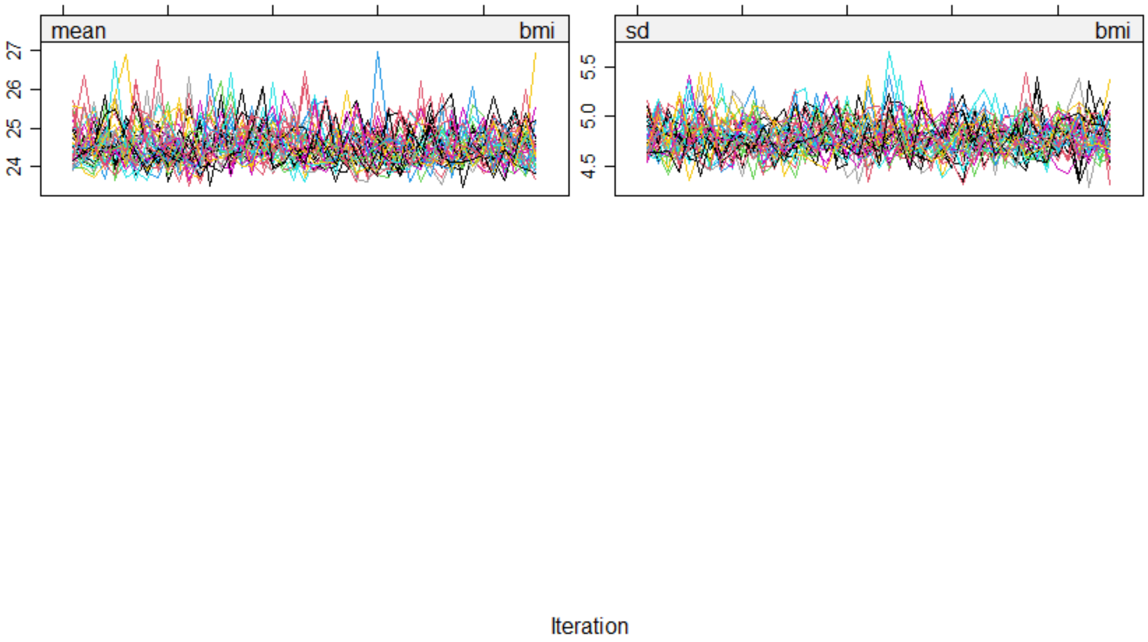

<u>The goodness of fit tests</u>

The model was considered fit.

1) The classification table was 70.5%.
2) The receiver operating curve (ROC) and the area under the curve (AUC):

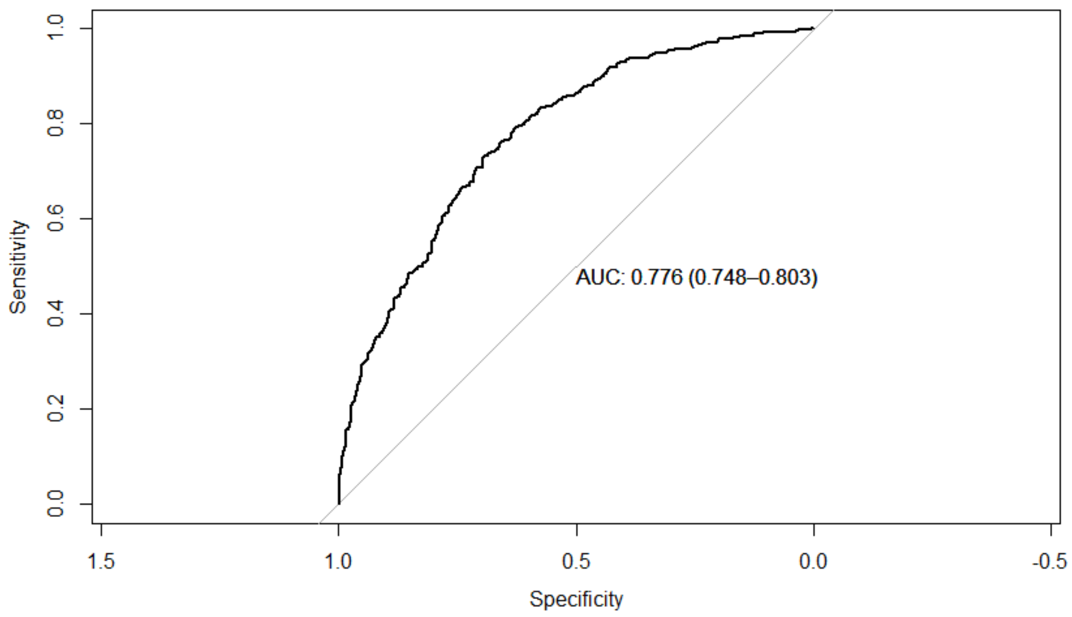

3) Hosmer-Lemeshow test was not significant (Chi-square = 10.23, df = 8, p = 0.249).
